# 14-Deoxy-11,12-didehydroandrographolide Promotes Ex Vivo Expansion of Umbilical Cord Blood Stem Cells through Stemness-Related Gene Regulation

**DOI:** 10.1021/acsomega.5c08975

**Published:** 2026-04-03

**Authors:** Laongthip Ruknarong, Nareerat Sutjarit, Nittaya Boonmuen, Puckjira Iaocharoen, Tippawan Duangsong, Wachirachai Pabuprapap, Apichart Suksamrarn, Duangrat Tantikanlayaporn

**Affiliations:** ¥ Center of Excellence in Stem Research and Innovation, Faculty of Medicine, 37699 Thammasat University, Pathum Thani 12120, Thailand; ψ Nutrition Unit, Faculty of Medicine Ramathibodi Hospital, Mahidol University, Bangkok 10400, Thailand; † Department of Physiology, Faculty of Science, 26687 Mahidol University, Bangkok 10400, Thailand; γ Department of Obstetrics and Gynecology, Faculty of Medicine, 37699 Thammasat University, Pathum Thani 12120, Thailand; # Department of Chemistry and Center of Excellence for Innovation in Chemistry, Faculty of Science, 54780 Ramkhamhaeng University, Bangkok 10240, Thailand; € Division of Cell Biology, Faculty of Medicine, 37699 Thammasat University, Pathum Thani 12120, Thailand

## Abstract

Umbilical cord blood (UCB) is a valuable alternative source of hematopoietic stem and progenitor cells (HSPCs) for allogeneic transplantation. However, the limited number of HSPCs that can be obtained from a single UCB unit remains a significant clinical challenge. Therefore, strategies to enhance the ex vivo expansion of functional HSPCs are of considerable interest. A key requirement for improving the success of HSC-based therapies, including transplantation and gene editing, is the ability to expand or preserve functional human HSCs during ex vivo culture. In this study, we demonstrate that 14-deoxy-11,12-didehydroandrographolide (14-DDA), a diterpenoid isolated from *Andrographis paniculata*, significantly promotes the ex vivo expansion of UCB-derived CD34^+^ cells and enriches for primitive HSPC subsets (CD34^+^CD38^–^CD90^+^). Functional assays reveal that 14-DDA enhances colony-forming unit output, which preserves multilineage differentiation potential. Furthermore, 14-DDA activates the Wnt/β-catenin signaling pathway, indicating a potential mechanism for supporting stem cell self-renewal. Gene expression profiling reveals upregulation of stemness- and proliferation-associated genes, while suppression of genes is related to differentiation, stress responses, and oncogenesis. These findings suggest that 14-DDA promotes the expansion of functional HSPCs while maintaining their primitive phenotype and reducing oncogenic risk, highlighting its potential as a natural small molecule for enhancing hematopoietic stem cell-based therapies.

## Graphical abstract

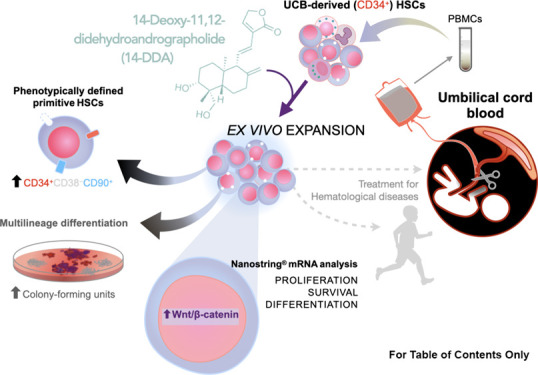

## Introduction

Umbilical cord blood (UCB) has emerged as a valuable alternative source of hematopoietic stem and progenitor cells (HSPCs) for allogeneic hematopoietic stem cell transplantation (HSCT), particularly for patients lacking matched bone marrow donors.^1^ UCB offers several advantages, including ease of collection, immediate availability, and a lower incidence of graft-versus-host disease (GVHD).^2^ However, the limited number of HSPCs in a single UCB unit poses a significant challenge, often leading to delayed engraftment and restricted use in adult recipients.^3^ To address this challenge, strategies to enhance the ex vivo expansion of UCB-derived HSPCs while preserving their self-renewal and multilineage differentiation capacity are of considerable interest in the field of regenerative medicine and transplantation.

Recent advances in the molecular regulation of hematopoietic stem and progenitor cell (HSPC) fate have highlighted the pivotal role of the Wnt/β-catenin signaling pathway in governing stem cell maintenance and expansion. Controlled activation of canonical Wnt signaling stabilizes β-catenin, leading to its nuclear translocation and activation of TCF/LEF transcriptional programs that reinforce self-renewal and block premature differentiation.^4^ Pharmacological strategies that enhance Wnt activity, such as glycogen synthase kinase-3β (GSK3β) inhibition by compounds like CHIR99021, have demonstrated efficacy in preserving primitive HSC pools during ex vivo culture and improving their long-term engraftment potential.^5,6^ More recently, plant-derived bioactive compounds have emerged as promising exogenous modulators of Wnt/β-catenin signaling. These natural molecules can fine-tune pathway activity, supporting HSPC expansion while preserving repopulating capacity and functional integrity.^7,8^ In addition, crosstalk between Wnt/β-catenin and other pathways, including PI3K/Akt, Notch, Hedgehog, and CXCL12-CXCR4, further refines the balance between quiescence and proliferation.^9^ Within this framework, both small molecules and plant-derived bioactive compounds are gaining increasing attention for their ability to influence the cellular signaling networks that govern stem cell proliferation and fate decisions.^8^

Among these, compounds derived from *Andrographis paniculata* (*A. paniculata*), a traditional medicinal plant widely used in Asia, have received considerable interest due to their diverse pharmacological activities, including immunomodulatory, anti-inflammatory, antioxidant, and anticancer effects.^10,11^ Recent studies suggest that these diterpenoids may influence fundamental stem cell behaviors, such as proliferation, differentiation, and maintenance of stemness through modulation of intracellular signaling pathways.^12,13^ Andrographolide, a major diterpenoid lactone derived from *A. paniculata*, has been reported to enhance osteogenic differentiation and promote the proliferative capacity of human mesenchymal stem cells through activation of the Wnt/β-catenin signaling pathway that also plays a pivotal role in hematopoietic stem cell (HSC) regulation.^14^ Interestingly, recent findings have highlighted the beneficial effects of andrographolide on the in vitro expansion of HSCs, suggesting its potential in stem cell-based applications.^15^ One of the major constituents of *A. paniculata*, 14-deoxy-11,12-didehydroandrographolide (14-DDA), has recently been shown to modulate redox homeostasis, autophagy, and cellular stress responses.^16^ While structurally related compounds such as andrographolide are known for their immunomodulatory, anti-inflammatory, and cytoprotective properties, the specific role of 14-DDA in hematopoiesis or stem cell biology remains largely unexplored.^17,18^ In this study, we investigated the effects of 14-DDA on the expansion and functional capacity of umbilical cord blood-derived hematopoietic stem and progenitor cells (UCB-HSPCs) and examined the molecular mechanisms underlying these effects. Our results indicate that 14-DDA promotes HSPC proliferation and enhances hematopoietic functionality through the modulation of key regulatory pathways. These findings suggest that 14-DDA represents a promising natural compound-based strategy to improve stem cell expansion protocols and may contribute to the development of more effective approaches for hematopoietic transplantation and regenerative therapies.

## Results and Discussion

### Isolation and Structural Identification of 14-DDA

14-Deoxy-11,12-didehydroandrographolide (14-DDA) was isolated as a white amorphous solid. The molecular formula of this compound was established as C_20_H_28_O_4_ by HR-TOFMS (positive ion electrospray ionization) at *m*/*z* 355.1889 [M + Na]^+^ (calcd for C_20_H_28_NaO_4_, 355.1880). The IR spectrum showed the presence of hydroxy groups (3279 cm^–1^), an α,β-unsaturated-γ-lactone (1748 and 1637 cm^–1^), and an exomethylene moiety (951 cm^–1^). The ^13^C NMR spectrum indicated the presence of a labdane-type skeleton with an exomethylene at δ 109.0 (C-17), a tertiary methyl at δ 22.6 (C-18), a hydroxymethyl at δ 64.0 (C-19), and a methyl signal at δ 15.8 (C-20). The ^1^H NMR spectrum also indicated an α,β-unsaturated γ-lactone moiety with three protons at δ 7.14 (t, *J* = 2.1 Hz, 1H, H-14) and 4.77 (dd, *J* = 19.0, 2.1 Hz, 2H, H-15) and corresponding ^13^C NMR signals at δ 129.1 (C-13), 143.1 (C-14), 69.7 (C-15), and 172.5 (C-16). The ^1^H NMR spectrum showed a carbinolic proton at δ 3.39 (dd, *J* = 11.1, 5.2 Hz) and the corresponding ^13^C NMR signal at δ 80.5, which was assigned to the hydroxyl group at the C-3 position. In addition, the ^1^H NMR spectrum exhibited signals of a 1,2-disubstituted *trans*-olefin at δ 6.80 (dd, *J* = 15.8, 10.1 Hz, 1H) and 6.06 (d, *J* = 15.8 Hz, 1H), assignable to H-11 and H-12, respectively. The chemical structure of 14-DDA is shown in Figure 1A, and the identity of this compound was confirmed by comparison of spectroscopic data and some physical data with those of the reported values.^19^ The spectroscopic spectra (^1^H and ^13^C NMR and HR-TOFMS) of 14-DDA are presented in the Supporting Information.

**Figure 1:**
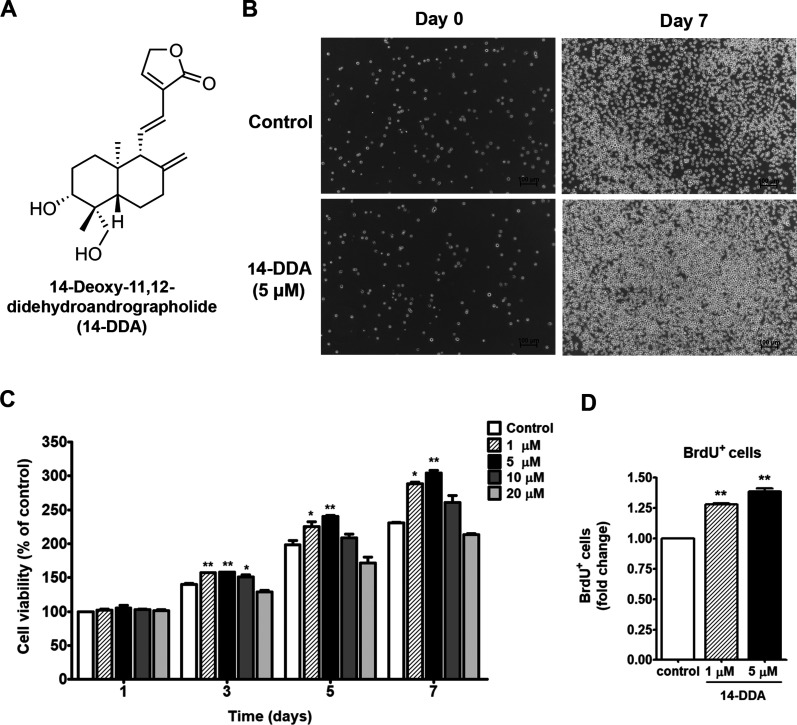
Effects of 14-DDA on the cell viability and proliferation of UCB-CD34^+^ cells. (A) Chemical structure of 14-DDA. (B) Representative images showing the morphological changes of cells before and after treatment with 14-DDA (5 μM) on days 0 and 7 (10× objective; scale bar, 100 μm). (C) Cell viability assessed following treatment with varying concentrations of 14-DDA on days 1, 3, 5, and 7. (D) Fold change of BrdU-positive (BrdU^+^) cells on day 7 after 14-DDA treatment. Results are presented as means ± SEM. Statistical significance was defined as *P < 0.05 and **P < 0.01 versus control.

### Effects of 14-DDA on the Cell Viability and Proliferation of UCB-CD34^+^ Cells

To assess the biological activity of 14-DDA, we examined its effects on the viability and proliferation of UCB-CD34^+^ cells during a 7-day culture period. Microscopic observations showed that cells treated with 5 μM 14-DDA exhibited markedly increased confluency by day 7 compared to the untreated control (Figure 1B), suggesting enhanced proliferative potential. Consistent with these morphological changes, cell viability assays revealed that 14-DDA promoted cell survival in a time- and dose-dependent manner (Figure 1C). While no significant difference was observed at day 1, a substantial increase in viability was evident from day 3 onward at concentrations ranging from 1–10 μM, with the most pronounced effect observed on days 5 and 7 (*P* < 0.01). Remarkably, treatment with 5 μM 14-DDA resulted in the highest cell viability on day 7, exceeding 250% compared to the control. However, concentrations above 10 μM resulted in reduced cell viability. Therefore, 14-DDA at 1 and 5 were selected for subsequent experiments.

To further validate the proliferative effect of 14-DDA, we performed BrdU incorporation assays, which confirmed a significant increase in DNA synthesis upon treatment (Figure 1D). Specifically, 1 and 5 μM of 14-DDA significantly elevated the proportion of BrdU^+^ cells compared to control cultures (*P* < 0.01), indicating stimulation of cell cycle progression. Collectively, these results demonstrate the proliferative activity of 14-DDA on UCB-derived HSPCs.

### Effect of 14-DDA on the Ex Vivo Expansion of Phenotypically Defined Primitive HSCs

To investigate the effect of 14-DDA on the expansion of primitive hematopoietic stem cells (HSCs), UCB-derived CD34^+^ cells were cultured in expansion medium containing 1 and 5 μM 14-DDA for 7 days. Flow cytometric analysis revealed that 14-DDA treatment significantly increased the fold change of CD34^+^ cells as well as primitive HSC subsets in a dose-dependent manner (Figure 2). Notably, fold expansion of CD34^+^CD90^+^CD34^+^CD38^–^ populations was markedly enhanced in both treatment groups compared to the control (*P* < 0.01). Furthermore, the most primitive subset, defined as CD34^+^CD38^–^CD90^+^, was also significantly enriched following treatment. These findings indicate that 14-DDA promotes the expansion or maintenance of primitive hematopoietic stem cell populations. These findings suggest that 14-DDA exerts a dual role in promoting both proliferation and the maintenance of stemness within the cultured HSC population.

**Figure 2:**
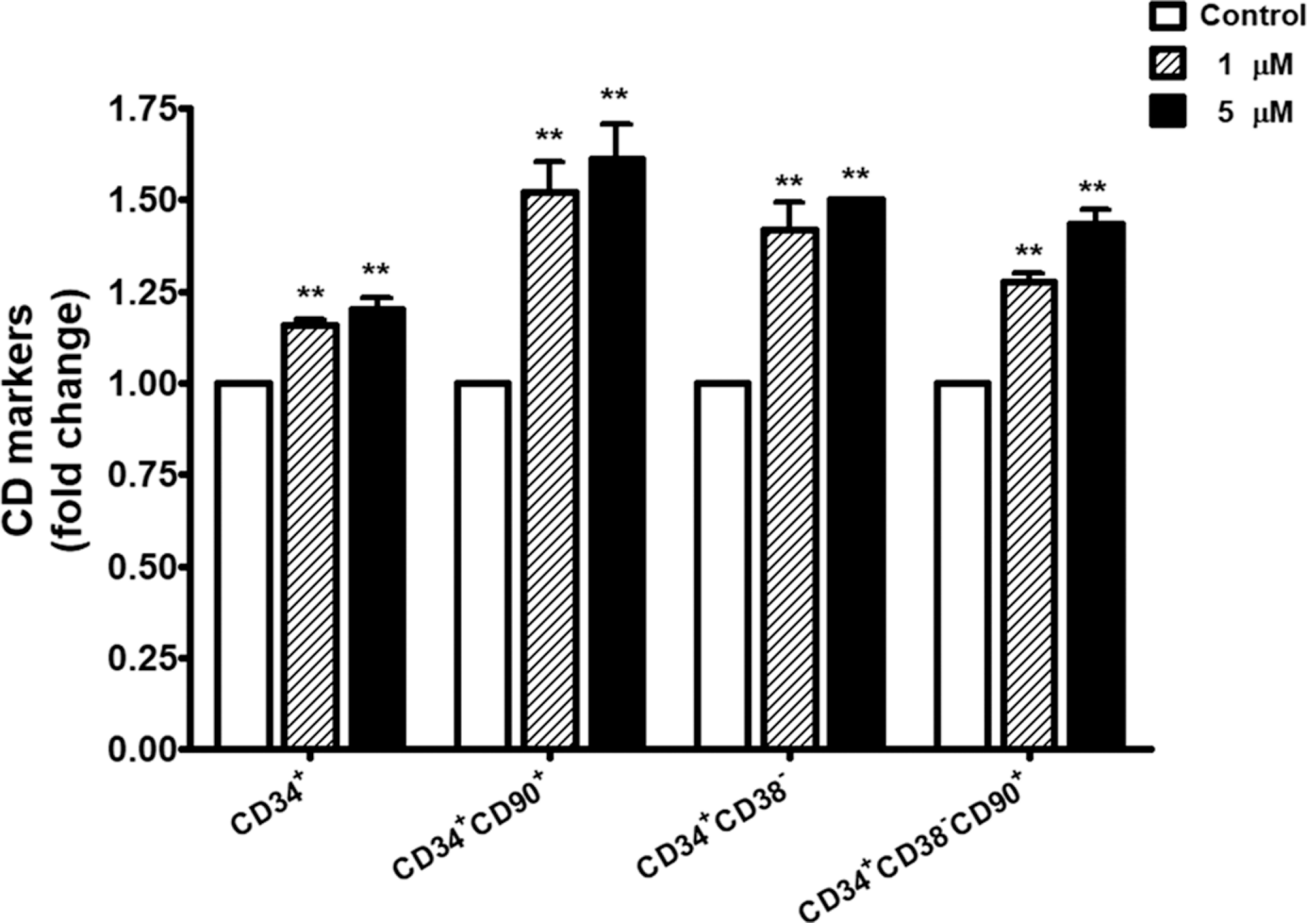
Effect of 14-DDA on the ex vivo expansion of phenotypically defined primitive HSC subsets. Representative flow cytometry plots showing the expression of primitive HSC markers after 7 days of culture in the presence or absence of 14-DDA (1 and 5 μM). The analyzed subpopulations include CD34^+^, CD34^+^CD90^+^, CD34^+^CD38^–^, and CD34^+^CD38^–^CD90^+^. Quantitative data are presented as means ± SEM from three independent experiments. Statistical significance was determined relative to the control group: *P < 0.05, **P < 0.01.

### 14-DDA Upregulates Stemness-Associated and HSC-Regulatory Genes

To investigate the molecular mechanisms by which 14-DDA supports hematopoietic stem cell (HSC) expansion, we analyzed the expression of genes associated with stemness and HSC regulation. Treatment with 14-DDA led to a dose-dependent upregulation of key surface markers and transcriptional regulators. As shown in Figure 3A, 14-DDA significantly increased the expression of *ALDH1A1*, *ALDH1A2*, *CD34*, *CD133*, and *CD117*, with the most pronounced induction observed at 5 μM (*P* < 0.01). Notably, *ALDH1A1* and *CD34* expression levels were elevated by more than 3-fold compared to the control, indicating a strong enhancement of stem cell-associated phenotypes. Furthermore, Figure 3B demonstrates that 14-DDA upregulated the transcription of critical HSC-regulatory genes, including *BMI1*, *HOXB4*, *RUNX1*, and *CXCR4*, with fold increases ranging from approximately 1.5 to over 3.0. These transcriptional changes suggest that 14-DDA may enhance the self-renewal capacity and preserve the undifferentiated state of primitive HSCs. Taken together, these findings provide strong molecular evidence that 14-DDA promotes the maintenance of stemness and functional properties of HSCs.

**Figure 3:**
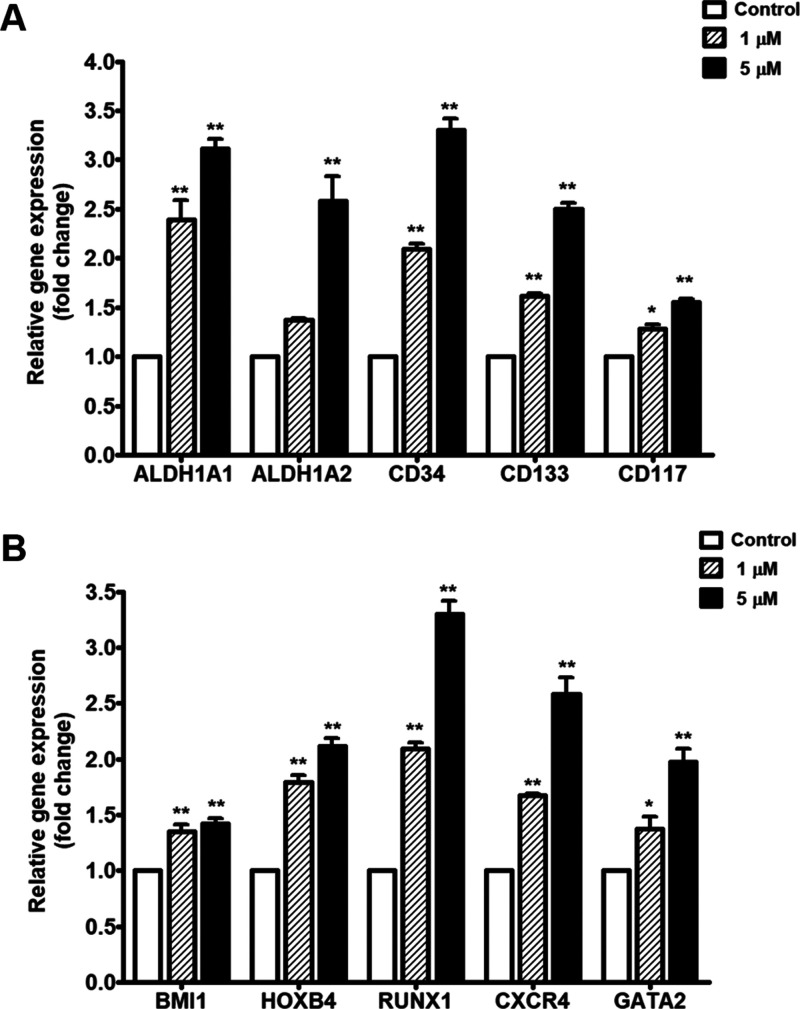
Effect of 14-DDA on the expression of characteristic genes related to HSC stemness. Relative gene expression levels are shown as a fold change after 7 days of culture with 14-DDA: (A) HSC-specific markers and functionally important genes (*ALDH1A1, ALDH1A2, CD34, CD133,* and *CD117*) and (B) HSC-relevant genes and transcription factors (*BMI1, HOXB4, RUNX1, CXCR4*, and *GATA2*). Data are presented as means ± SEM from three independent experiments. *P < 0.05 and **P < 0.01 compared with the control.

### 14-DDA Sustains Multipotency of HSC In Vitro

A major limitation in ex vivo expansion protocols for HSPCs is the potential loss of their multipotent differentiation capacity. To evaluate whether 14-DDA-treated UCB-CD34^+^ cells retained multilineage potential, colony-forming unit (CFU) assays were performed after 14 days of culture in methylcellulose medium. Distinct hematopoietic progenitor colonies, including burst-forming unit erythroid (BFU-E), colony-forming unit-granulocyte-macrophage (CFU-GM), and colony-forming unit-granulocyte-erythrocyte-macrophage-megakaryocyte (CFU-GEMM), were enumerated and morphologically assessed. As shown in Figure 4A, the colony morphology and size were comparable between 14-DDA-treated and control groups, indicating that 14-DDA does not exert cytotoxic effects on progenitor differentiation. Quantitative analysis (Figure 4B) revealed that BFU-E colonies remained abundant, while CFU-GM and CFU-GEMM colonies were slightly increased, suggesting that 14-DDA not only preserves multipotency but may also enhance the proliferative and lineage commitment potential of hematopoietic progenitors.

**Figure 4:**
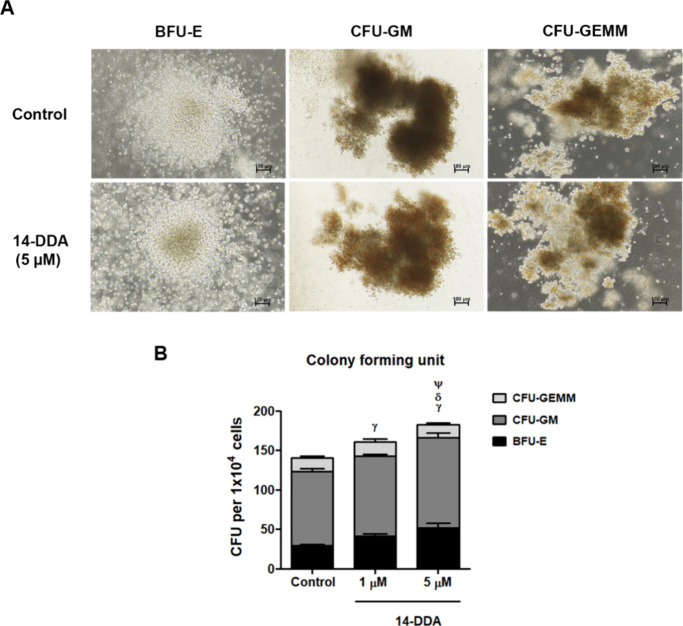
Colony-forming capacity of UCB-CD34^+^ cells expanded with 14-DDA. (A) Representative images of hematopoietic colonies: BFU-E (burst-forming unit erythroid), CFU-GM (colony-forming unit-granulocyte-macrophage), and CFU-GEMM (colony-forming unit-granulocyte-erythrocyte-macrophage-megakaryocyte) after 14 days of culture in methylcellulose-based medium supplemented with cytokines alone or with 5 μM 14-DDA. (B) Quantitative analysis of colony numbers based on size and cell morphology. Data are presented as the mean ± SEM from three independent experiments. Statistical significance compared to the control group is denoted as *P < 0.05 and **P < 0.01. Comparing each colony between groups: γ represents the BFU-E colony, δ represents the CFU-GM colony, and ψ represents the CFU-GEMM colony. Data are represented as means ± SEM from three independent experiments. γ: *P* < 0.05, δ: *P* < 0.05, and ψ: *P* < 0.05, compared with the control group for each colony.

### Modulatory Effects of 14-DDA on Wnt/β-Catenin Signaling Pathways in UCB-CD34^+^ Cells

To further explore the molecular mechanisms underlying the effects of 14-DDA on HSC expansion, we assessed the expression of genes involved in the canonical Wnt/β-catenin signaling pathway, a key regulator of stem cell self-renewal and proliferation. Treatment of UCB-CD34^+^ cells with 14-DDA significantly upregulated the expression of *CTNNB1* (β-catenin), the central effector of this signaling cascade. In addition, 14-DDA markedly induced the expression of downstream β-catenin target genes, including *LEF1*, *FZD2*, *CCND1*, *C-MYC*, and *AXIN2* (Figure 5). These findings suggest that 14-DDA may modulate the Wnt/β-catenin pathway to promote HSC maintenance and expansion.

**Figure 5:**
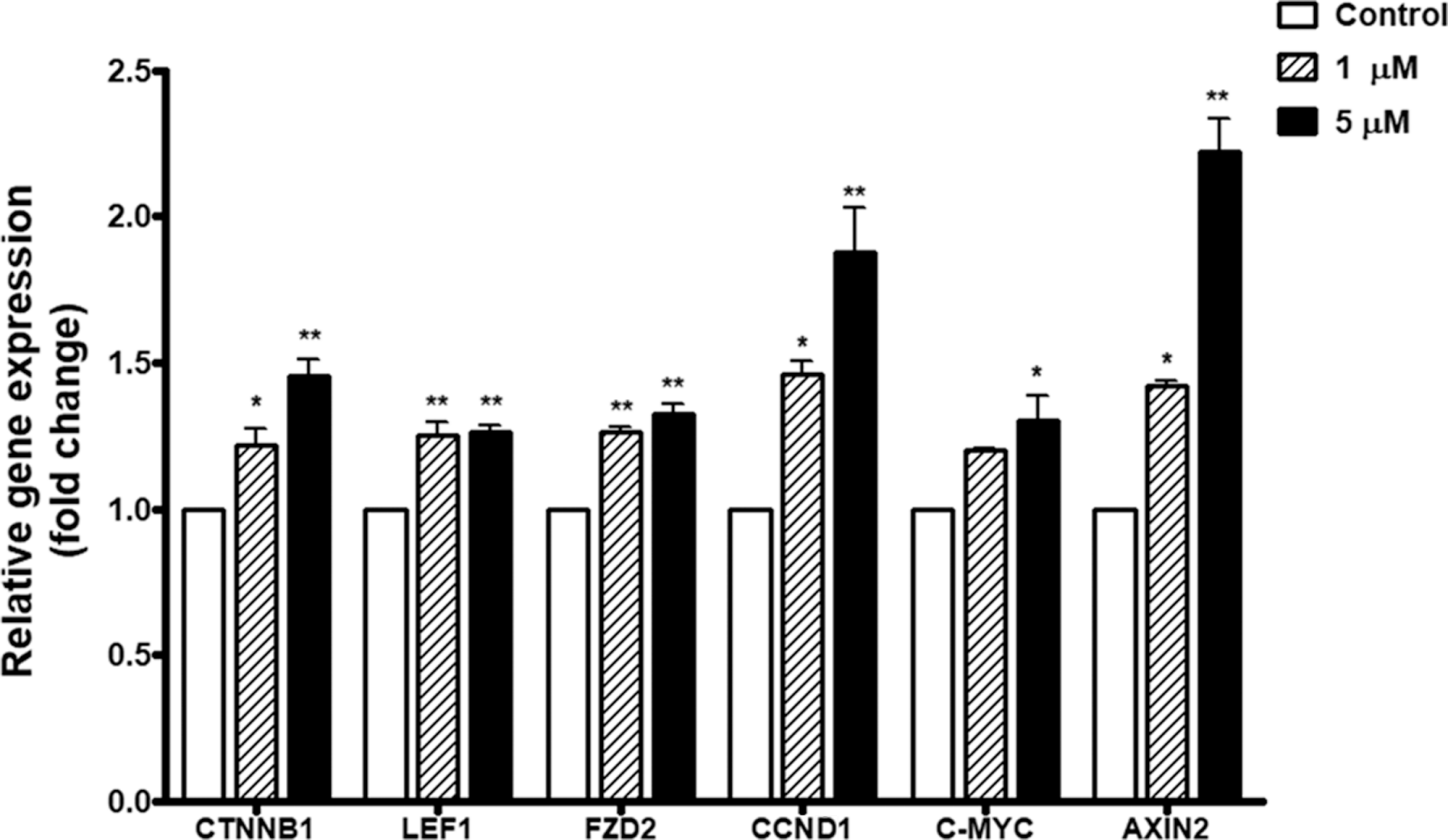
Effect of 14-DDA on gene expression of Wnt/β-catenin-associated genes, including *CTNNB1*, *LEF1*, *FZD2*, *CCND1*, *C-MYC*, and *AXIN2*, in UCB-CD34^+^ cells. Data represent the mean fold changes in gene expression in 14-DDA-treated cells relative to the control. *P < 0.05 and **P < 0.01 compared to the control.

### Molecular Effects of 14-DDA on Gene Expression in CD34^+^ Cells

To investigate the molecular effects of 14-DDA on the expansion of umbilical cord-derived hematopoietic stem cells (UC-HSCs), NanoString gene expression profiling was performed. The analysis revealed that 14-DDA modulates a diverse set of genes associated with cell proliferation, stemness, angiogenesis, extracellular matrix remodeling, cell adhesion and migration, stress responses, and oncogenesis (Figure 6 and Table 1). Notably, 14-DDA treatment upregulated several genes critical for maintaining stem cell properties and enhancing cell proliferation, including *VEGFA*, a key angiogenic factor; *EPCAM*, a surface marker linked to progenitor and stem-like characteristics; and *PIM1*, *MYC*, and *FOSL2*, all of which are well-established regulators of cellular growth and survival.

**Figure 6:**
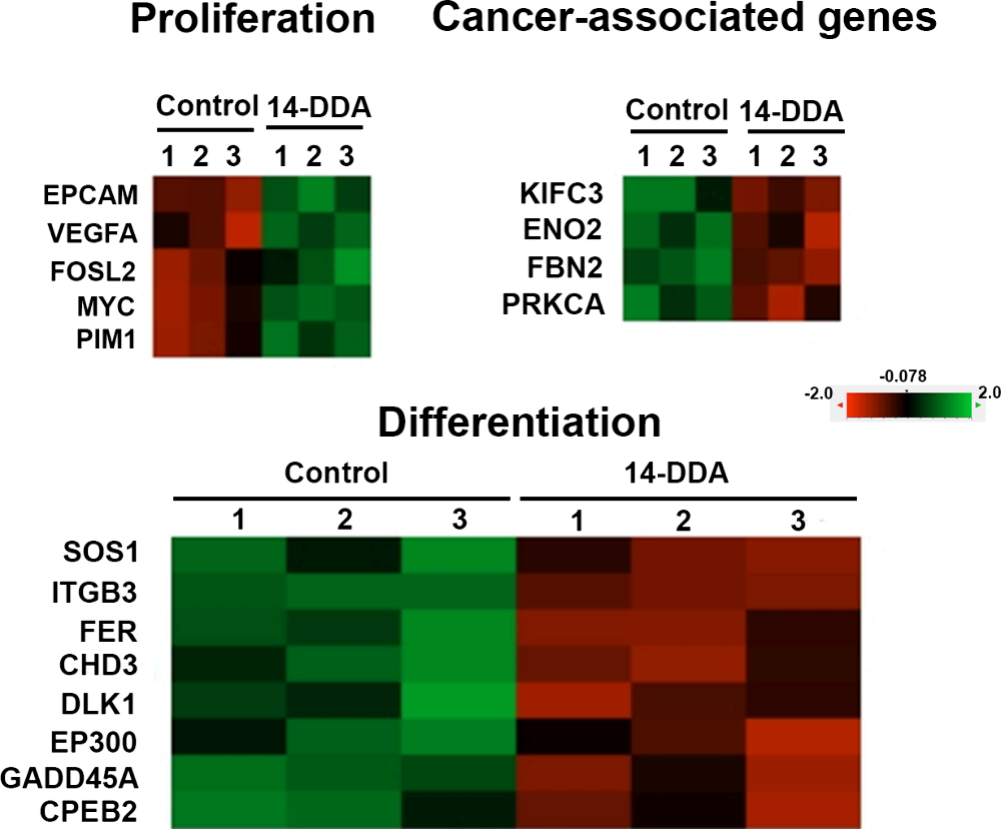
Molecular analysis in expanded UCB-CD34^+^ cells treated with 14-DDA. Heat map showing the differential expression of candidate genes in expanded UCB-CD34^+^ cells treated with 5 μM 14-DDA compared to control cells on day 7. Total mRNA was isolated from triplicate samples and analyzed using the NanoString analysis system. Genes with *P* < 0.05 were considered significantly differentially expressed.

**Table 1:** List of Significantly Altered Genes (*P* < 0.05) by 14-DDA Treatment Identified via NanoString Analysis

gene symbol	gene name	fold change	*P* value	gene ID
Proliferation				
VEGFA	vascular endothelial growth factor A	1.14	0.0309	NM_001025366.1
EPCAM	epithelial cell adhesion molecule	1.1	0.0032	NM_002354.2
PIM1	Pim1 serine/threonine kinase	1.14	0.0148	NM_002648.3
FOSL2	FOS-like antigen 2	1.12	0.0338	NM_005253.3
MYC	MYC	1.16	0.0232	NM_002467.3
Differentiation				
CHD3	chromodomain helicase DNA-binding protein 3	–1.14	0.0120	NM_001005271.2
EP300	E1A binding protein p300	–1.13	0.0395	NM_001429.2
FER	FER tyrosine kinase	–1.16	0.0058	NM_005246.1
SOS1	son of sevenless homologue 1	–1.1	0.0207	NM_005633.2
CPEB2	cytoplasmic polyadenylation element binding protein 2	–1.14	0.0214	NM_001177381.1
GADD45A	growth arrest and DNA damage-inducible alpha	–1.12	0.0165	NM_001199741.1
DLK1	delta-like noncanonical Notch ligand 1	–1.1	0.0321	NM_001317172.1
ITGB3	integrin, beta 3	–1.12	0.0002	NM_000212.2
Cancer-associated genes				
ENO2	enolase 2	–1.17	0.0226	NM_001975.2
FBN2	fibrillin 2	–1.18	0.0020	NM_001999.3
KIFC3	kinesin family member C3	–1.21	0.0173	NM_001130099.1
PRKCA	protein kinase C, alpha	–1.15	0.0135	NM_002737.2

In contrast, 14-DDA downregulated the expression of multiple genes implicated in differentiation, stress signaling, and tumor progression. These include fate decisions and differentiation genes such as *EP300*, *CHD3*, *FER*, and *SOS1*; adhesion- and migration-related genes such as *APBB1IP*, *ITGB3*, and *NID2*; and stress response genes including *GADD45A*, *DLK1*, and *CPEB2*. Importantly, 14-DDA also suppressed several cancer-associated genes, including *ENO2*, *FBN2*, *KIFC3*, and *PRKCA*, which are involved in tumor metabolism, extracellular matrix remodeling, mitotic control, and oncogenic signaling. Together, these findings suggest that 14-DDA induces a transcriptional program in CD34^+^ cells that favor proliferation and maintenance of stemness while concurrently inhibiting pathways related to differentiation, cell motility, and cancer-related gene expression.

## Discussion

A key requirement for improving the success of HSC-based therapies, including transplantation and gene editing, is the ability to expand or preserve functional human HSCs during ex vivo culture.^20,21^ Various strategies have been developed to optimize culture conditions that support both HSC proliferation and the maintenance of pluripotency.^22^ In this study, we demonstrated for the first time that 14-DDA significantly enhances the ex vivo expansion of UCB-CD34^+^ cells. In addition to promoting expansion, 14-DDA preserved the functional integrity of these cells, including their self-renewal capacity and ability to differentiate into multiple hematopoietic lineages. These effects are likely mediated through coordinated regulation of critical signaling pathways, notably Wnt/β-catenin, which play fundamental roles in maintaining HSC stemness, supporting proliferation, and inhibiting premature differentiation.

Our study demonstrates that 14-DDA exerts a proliferative effect on hematopoietic cells, as evidenced by enhanced cell viability. These findings are further corroborated by morphological analysis and BrdU incorporation, which consistently support the stimulatory effect of 14-DDA on cell growth. In addition, 14-DDA not only enhanced cell proliferation but also contributed to the expansion of primitive hematopoietic stem cells (HSCs), as reflected by a significant increase in the CD34^+^CD38^–^CD90^+^CD45RA^–^ population in expanded UCB-CD34^+^ cells compared to the control. This suggests that 14-DDA may help maintain stemness while promoting proliferation, a dual effect that is particularly beneficial for ex vivo HSC expansion.^23^ Collectively, these results highlight the potential of 14-DDA as a promising small molecule for supporting stem cell proliferation and preservation of primitive HSC phenotypes in vitro.

Moreover, the upregulation of *ALDH1A1*, *CD34*, *CD133*, and *CD117* genes by 14-DDA supports its role in maintaining a primitive hematopoietic phenotype. ALDH activity is a recognized marker of long-term repopulating HSCs,^24^ while CD34 and CD133 are commonly used for HSC enrichment.^25^ The induction of HOXB4 and BMI1, critical transcription factors for HSC self-renewal, reinforces their function in preserving HSC characteristics.^26,27^ Functional validation by the colony-forming unit (CFU) assay confirmed these transcriptional effects. The increase in BFU-E and CFU-GM colonies indicates stimulation of erythroid and myeloid progenitors, respectively, while a significant rise in CFU-GEMM colonies, derived from multipotent progenitors, suggests that 14-DDA supports the maintenance or expansion of early hematopoietic progenitors.^28^ Together, these findings highlight 14-DDA as a promising small molecule for ex vivo HSPC expansion or preconditioning strategies in hematopoietic transplantation.

The Wnt/β-catenin pathway plays a crucial role in regulating adult stem cell fate, particularly in sustaining self-renewal and inhibiting premature differentiation of HSCs.^29,30^ The Wnt signaling pathway primarily exerts its effects through β-catenin, a key regulator known to enhance HSC self-renewal and proliferation. Activation of *CTNNB1* (β-catenin) by 14-DDA, as observed in our study, along with the upregulation of *LEF1* and *FZD2*, suggests stimulation at both the receptor level and the transcriptional components of the Wnt/β-catenin signaling pathway.^30,31^ The significant increases in *CCND1* and *C-MYC* expression further support the role of 14-DDA in enhancing cell cycle progression and proliferative capacity.^32,33^ Additionally, the upregulation of *AXIN2*, a feedback inhibitor and hallmark indicator of Wnt pathway activity,^34^ reinforces the conclusion that 14-DDA actively engages this signaling axis. Taken together, these results suggest that activation of the Wnt/β-catenin signaling pathway is a key mechanism by which 14-DDA supports UCB-CD34^+^ cell expansion.

Previous studies have shown that modulating specific signaling pathways, either through activation or inhibition, can significantly influence HSC proliferation,^35^ thereby affecting both engraftment efficiency and in vitro expansion.^36^ In this study, we employed NanoString gene expression profiling to elucidate the molecular mechanisms by which 14-DDA promotes the expansion of UC-HSCs. Our results demonstrate that 14-DDA exerts a broad regulatory effect on genes involved in key cellular processes such as proliferation, stemness maintenance, angiogenesis, extracellular matrix remodeling, adhesion, migration, stress response, and oncogenesis.

Specifically, 14-DDA significantly upregulated genes critical for sustaining HSC properties and enhancing proliferation, including *VEGFA*, a potent angiogenic factor known to support the hematopoietic niche; *EPCAM*, associated with progenitor and stem-like cell phenotypes; and oncogenes such as *PIM1*, *MYC*, and *FOSL2*, which are established mediators of cell growth and survival.^37–39^ This gene expression pattern aligns well with the observed phenotypic expansion and preservation of primitive HSC markers, suggesting that 14-DDA reinforces self-renewal capacity and proliferative potential through activation of these pathways. Conversely, 14-DDA downregulated multiple genes implicated in differentiation, stress signaling, and tumor progression. Among these, *EP300*, *CHD3*, and *CSNK1A1* are known to influence chromatin remodeling and differentiation, while *FER* and *SOS1* regulate intracellular signaling cascades related to cell fate decisions.^40–42^ The suppression of adhesion- and migration-related genes such as *APBB1IP*, *ITGB3*, and *NID2* may contribute to maintaining a more quiescent and less migratory stem cell phenotype, which is favorable for ex vivo expansion. Furthermore, the downregulation of stress response genes, including *GADD45A* and *DLK1*, suggests a reduction in cellular stress and DNA damage signaling, which could promote cell survival during culture.^43^

Importantly, 14-DDA also suppressed several cancer-associated genes involved in tumor metabolism (*ENO2*),^44^ extracellular matrix remodeling (*FBN2*),^45^ mitotic control (*KIFC3*),^46^ and oncogenic signaling (*PRKCA*).^47^ This finding is particularly relevant for the clinical application of expanded HSCs, as it indicates that 14-DDA not only enhances stemness and proliferation but also mitigates oncogenic risks that might arise during ex vivo culture.

Our findings suggest that 14-DDA promotes ex vivo expansion and preserves primitive HSCs, but important limitations remain. While in vitro assays support its role in maintaining stemness and proliferation, in vivo validation through long-term repopulation and engraftment studies is required to confirm clinical relevance. Moreover, its involvement in Wnt/β-catenin and related pathways is inferred from transcriptional data and warrants mechanistic validation using pathway-specific interventions. Nevertheless, these results provide preliminary evidence that 14-DDA drives a transcriptional program favoring proliferation and stemness while restraining differentiation, migration, and oncogenic signaling, highlighting its potential to enhance HSC-based therapies.

## Conclusions

In conclusion, our study demonstrates that 14-DDA promotes the ex vivo expansion of primitive UC-HSCs by enhancing proliferation and maintaining stemness-associated characteristics. This effect appears to involve modulation of key signaling pathways and gene networks that support self-renewal while inhibiting differentiation and oncogenic programs. Collectively, these findings suggest that 14-DDA holds promise for enhancing HSC-based therapies.

## Methods

### Ethics Approval and Consent to Participate

All procedures involving human umbilical cord blood (UCB) were reviewed and approved by the Human Research Ethics Committee of Thammasat University (Faculty of Medicine), under protocol number COA 035/2023; MTU-EC-DS-1–260/65, with approval granted on 25 January 2023. The study was conducted in full compliance with the Declaration of Helsinki, the Belmont Report, and ICH-GCP guidelines. Prior to sample collection, each donor received a participant information sheet and provided written informed consent. All experimental methods adhered to applicable ethical and regulatory standards. Fresh human umbilical cord blood (UCB) samples, collected within 2–4 h, were obtained from Thammasat Chalermprakiet Hospital, Thammasat University. Ethical approval for all experimental procedures was granted by the Human Research Ethics Committee of Thammasat University (Faculty of Medicine), in accordance with the principles outlined in the Declaration of Helsinki.

### UCB-CD34^+^ Cell Isolation and In Vitro Culture

CD34^+^ cells were isolated from freshly collected whole umbilical cord blood using a simple two-step protocol. Initially, hematopoietic progenitor cells were enriched using the RosetteSep Human Cord Blood CD34^+^ Pre-Enrichment Cocktail (STEMCELL Technologies, Canada). This was followed by magnetic separation of CD34^+^ cells using the EasySep Human CD34 Positive Selection Kit for umbilical cord blood, performed in accordance with the manufacturer’s instructions (STEMCELL Technologies, Canada).

To assess cell purity, isolated cells were labeled with an APC-conjugated antihuman CD34 antibody (BD Biosciences; 555824) in phosphate-buffered saline (PBS) and incubated at 4 °C for 30 min. After washing with PBS, samples were analyzed using a DX Flex flow cytometer (Beckman Coulter). Flow cytometric analysis confirmed that the purity of CD34^+^ cells exceeded 95%. For in vitro expansion, purified UCB-derived CD34^+^ cells were cultured in StemSpan Expansion Medium (STEMCELL Technologies, Canada) supplemented with 50 ng/mL stem cell factor (SCF), 50 ng/mL thrombopoietin (TPO), 20 ng/mL FLT-3 ligand (Flt-3L), 20 ng/mL interleukin-6 (IL-6), 20 ng/mL soluble IL-6 receptor (sIL-6R) (all from PeproTech, USA), and 1% (v/v) penicillin/streptomycin (P/S) (Merck Millipore, USA). Cultures were maintained at 37 °C in a humidified incubator with 5% CO_2_ in air.

### Isolation of 14-Deoxy-11,12-didehydroandrographolide (14-DDA)

#### General Experimental Procedures

IR spectra were recorded in the ATR mode using a PerkinElmer FT-IR Spectrum 400 spectrophotometer. ^1^H and ^13^C NMR spectra were recorded on a Bruker ASCEND 400 FT-NMR spectrometer, operating at 400 and 100 MHz for ^1^H and ^13^C NMR, respectively. HR-TOFMS spectra were measured with a Bruker micrOTOF-QII mass spectrometer, using electrospray ionization (ESI) as the ionization technique. Column chromatography was carried out using Merck silica gel 60 (particle sizes <0.063 mm). For thin-layer chromatography (TLC), Merck precoated silica gel 60 F_254_ plates were used. TLC spots were detected under UV light and by spraying with an anisaldehyde-H_2_SO_4_ reagent followed by heating.

14-DDA was isolated from the dried aerial parts of *Andrographis paniculata*, which were harvested in September 2021 from Prachantakham District, Prachin Buri Province, Thailand. Briefly, the plant material was milled into small pieces and successively macerated with *n*-hexane and CH_2_Cl_2_ at room temperature, with three replications for each solvent. The solvents were then removed under reduced pressure to yield hexane and CH_2_Cl_2_ extracts. The crude CH_2_Cl_2_ extract was subjected to column chromatography, and the fractions containing 14-DDA by TLC investigation were combined. Crystallization from EtOAc gave 14-DDA, which upon recrystallization from EtOAc furnished pure 14-DDA. The purity of 14-DDA was >95% as determined by TLC and NMR investigations.

#### 14-Deoxy-11,12-didehydroandrographolide (14-DDA)

White amorphous solid (from EtOAc); mp. 186–187 °C; ${\left[\alpha \right]}_{D}^{25}$ −3.13 (*c* 0.48, MeOH); IR (ATR): ν_max_ 3279, 2932, 2856, 1748, 1637, 1439, 1350, 1207, 1091, 1038, 951 cm^–1^; ^1^H NMR (400 MHz, CDCl_3_ + 5 drops CD_3_OD): δ 7.14 (t, *J* = 2.1 Hz, 1H, H-14), 6.80 (dd, *J* = 15.8, 10.1 Hz, 1H, H-11), 6.06 (d, *J* = 15.8 Hz, 1H, H-12), 4.77 (dd, *J* = 19.0, 2.1 Hz, 2H, H-15), 4.72 (brs, 1H, H-17b), 4.47 (brs, 1H, H-17a), 4.15 (d, *J* = 11.1 Hz, 1H, H-19b), 3.39 (dd, *J* = 11.1, 5.2 Hz, 1H, H-3), 3.28 (d, *J* = 11.1 Hz, 1H, H-19a), 2.40 (ddd, *J* = 13.6, 4.3, 2.3 Hz, 1H, H-7b), 2.26 (brd, *J* = 10.1 Hz, 1H, H-9), 2.01–1.98 (m, 1H, H-7a), 1.74–1.68 (m, 3H, H-2, H-6b), 1.45 (brt, *J* = 13.6 Hz, 1H, H-1b), 1.29 (ddd, *J* = 17.2, 12.9, 4.3 Hz, 1H, H-6a), 1.20 (s, 3H, H-18), 1.19–1.07 (m, 2H, H-1a, H-5), 0.76 (s, 3H, H-20). ^13^C NMR (CDCl_3_ + 5 drops CD_3_OD, 100 MHz): δ 172.5 (C-16), 148.1 (C-8), 143.1 (C-14), 135.9 (C-11), 129.1 (C-13), 121.0 (C-12), 109.0 (C-17), 80.5 (C-3), 69.7 (C-15), 64.0 (C-19), 61.6 (C-9), 54.6 (C-5), 42.7 (C-4), 38.5 (C-10), 38.2 (C-1), 36.5 (C-7), 27.8 (C-2), 22.9 (C-6), 22.6 (C-18), 15.8 (C-20); HR-TOFMS (ESI+) *m*/*z* 355.1889 [M + Na]^+^ (calcd for C_20_H_28_NaO_4_, 355.1880).

### Cell Viability Assay

To determine the optimal concentration of 14-DDA for enhancing the viability of UCB-derived CD34^+^ cells, an MTS assay was performed. Cells were plated at 1000 cells per well in 96-well plates and treated with varying concentrations of 14-DDA (Sigma-Aldrich, St. Louis, MO, USA; see Figure 1A) for 1, 3, 5, and 7 days. Control groups received culture medium containing 0.01% DMSO as a vehicle. At each designated time point, 100 μL of the MTS reagent (Promega) was added to each well and incubated at 37 °C in the dark for 4 h. Absorbance was measured at 490 nm using a microplate reader. All measurements were normalized against the DMSO control group, and data are presented as the mean ± SEM from three independent experiments.

### BrdU Cell Proliferation Assay

To evaluate the proliferation of cultured UCB-derived CD34^+^ cells, the FITC BrdU Flow Kit (BD Biosciences) was utilized in conjunction with flow cytometry. Briefly, cells were initially fixed in Cytofix/Cytoperm Buffer and subsequently permeabilized using Cytoperm Permeabilization Buffer Plus. After centrifugation at 300*g* for 6 min, the cell pellets were incubated with DNase (300 μg/mL) to denature DNA and allow BrdU exposure. Following a 1 h incubation at 37 °C in the dark, cells were washed with BD Perm/Wash Buffer. To detect BrdU incorporation, cells were stained with a fluorescently tagged anti-BrdU antibody diluted in Perm/Wash Buffer and incubated at room temperature for 20 min in the dark. Samples were then washed once more and analyzed using a DX Flex flow cytometer (Beckman Coulter). The percentage of cells incorporating BrdU was used as a measure of proliferation.

### Immunophenotypic Analysis in Expanded Cells

For immunophenotypic analysis, expanded cells were stained at 4 °C for 30–60 min in PBS containing fluorophore-conjugated monoclonal antibodies: APC-conjugated antihuman CD34 (BD Biosciences; 555824), PE-Cy7-conjugated antihuman CD38 (BD; 560677), and BV421-conjugated antihuman CD90 (BD; 562556). Flow cytometric acquisition was performed using a DX Flex cytometer (Beckman Coulter), with at least 10^4^ events collected per sample. The resulting data were analyzed to determine surface marker expression in the CD34^+^ cell population following expansion.

### Colony-Forming Unit (CFU) Assay

UCB-derived CD34^+^ cells were cultured for 7 days in expansion medium supplemented with either 2.5 or 5 μM 14-deoxy-11,12-didehydroandrographolide (14-DDA). Following treatment, CD34^+^ cells were reisolated using magnetic microbeads targeting CD34 (STEMCELL Technologies). A total of 500 purified cells were seeded into a methylcellulose-based medium (H4434; STEMCELL Technologies, Canada) enriched with a 50 ng/mL stem cell factor (SCF), a 10 ng/mL granulocyte-macrophage colony-stimulating factor (GM-CSF), 10 ng/mL interleukin-3 (IL-3), and 3 IU/mL erythropoietin (EPO), with or without additional 14-DDA supplementation. Cells were incubated for 14 days, after which hematopoietic colonies were classified and counted under a light microscope. The colony types identified included burst-forming unit erythroid (BFU-E), colony-forming unit granulocyte-macrophage (CFU-GM), and colony-forming unit granulocyte-erythrocyte-macrophage-megakaryocyte (CFU-GEMM). Only colonies comprising more than 100 cells were considered for quantitative analysis.

### RNA Extraction and Quantitative RT-QPCR Analysis

Total RNA was isolated from UCB-derived CD34^+^ cells using an RNeasy Mini Kit (Qiagen, CA, USA) according to the manufacturer’s protocol. RNA concentration and purity were evaluated using a NanoDrop ND-1000 spectrophotometer (Thermo Fisher Scientific, USA). Subsequently, 500 ng of total RNA was reverse-transcribed into cDNA using an iScript Select cDNA Synthesis Kit (Bio-Rad Laboratories, Inc., USA). Quantitative real-time PCR (qRT-qPCR) was performed using an iTaq Universal SYBR Green Supermix (Bio-Rad Laboratories, Inc., USA) on an ABI StepOnePlus Real-Time PCR System (Applied Biosystems, CA, USA). Gene expression levels were calculated using the comparative Ct (2^–ΔΔCt^) method. GAPDH served as the internal reference gene, and relative gene expression levels are presented as fold changes normalized to GAPDH. Primer sequences used in this analysis are listed in Table 2.

**Table 2:** Sequences of Primers for Quantitative RT-qPCR Analyses

gene	forward (5′–3′)	reverse (5′–3′)
*CD34*	GGAAGGATGCTGGTCCG	CTGGGGTAGCAGTACCGTTG
*CD133*	CCATTGGCATTCTCTTTGAA	TTTGGATTCATATGCCTTCTGT
*CD117*	TGGGATTTTCTCTGCGTTCT	TGATTTTCCTGGATGGATGG
*ALDH1*	AATGGCATGATTCAGTGAGTGGC	GAGGAGTTTGCTCTGCTGGTTTG
*ALDH2*	CAAGATAGAGATGCCCGGCG	ACAGGGAACACTCTCCCACT
*BMI1*	CTTTCATTGTCTTTTCCGCC	TCCACAAAGCACACACATCA
*HOXB4*	CCTGGATGCGCAAAGTTCA	AATTCCTTCTCCAGCTCCAAGA
*RUNX1*	CGATGGCTTCAGACAGCATA	GGCATCGTGGACGTCTCTA
*GATA2*	CACAAGATGAATGGGCAGAA	ACAATTTGCACAACAGGTGC
*CXCR4*	CAGCAGGTAGCAAAGTGACG	CCCATTTCCTCGGTGTAGTT
*CTNNB1*	GCTTGTTCGTGCACATCAGGA	TGTGAACATCCCGAGCTAGGA
*AXIN2*	CCTGGCTCCAGAAGATCACA	AGCATCCTCCGGTATGGAAT
*LEF1*	GCCAGACAAGCACAAACCTCT	TGGCATCATTATGTACCCGG
*FZD2*	GTCCTCAAGGTGCCATCCTATC	GCGTCCCTCCTGTGAGAAGAA
*CCND1*	GATCAAGTGTGACCCGGACTG	CCTTGGGGTCCATGTTCTGC
*C-MYC*	ATGGCCCATTACAAAGCCG	TTTCTGGAGTAGCAGCTCCTAA
*GAPDH*	GAGTCAACGGATTTGGTCGT	TTGATTTTGGAGGGATCTCG

### NanoString Analysis

Gene expression profiling was performed using the NanoString nCounter Analysis System, which utilizes digital barcoding technology for direct multiplex quantification of mRNA transcripts. A total of 100 ng of RNA per sample was hybridized with probes targeting 770 genes included in the nCounter Stem Cells Panel (NanoString Technologies), following the manufacturer’s protocol. Hybridized samples were processed using the NanoString Prep Station and scanned on the nCounter Digital Analyzer. Results were normalized using 29 internal reference (housekeeping) genes, and all heat maps were generated using the advanced analysis packages in nSolver 4.0 NanoString nSolver software.

### Statistical Analysis

All data are presented as means ± standard error of the mean (SEM). Statistical comparisons among multiple groups were performed using one-way analysis of variance (ANOVA), followed by Bonferroni's multiple-comparison test. Analyses were conducted using GraphPad Prism software, version 9.0 for Windows (GraphPad Software, San Diego, CA, USA). A *P* value of less than 0.05 was considered to indicate statistical significance.

## Supplementary Material



## Notes

^1^H NMR spectrum (400 MHz) of 14-deoxy-11,12-didehydroandrographolide (14-DDA) in CDCl_3_ + 5 drops CD_3_OD (Figure S1), ^13^C NMR spectrum (100 MHz) of 14-deoxy-11,12-didehydroandrographolide (14-DDA) in CDCl_3_ + 5 drops CD_3_OD (Figure S2), and HR-TOFMS (ES^+^) spectrum of 14-deoxy-11,12-didehydroandrographolide (14-DDA) (Figure S3) (PDF)
