# Supplementary material for: 14-Deoxy-11,12-didehydroandrographolide Promotes Ex Vivo Expansion of Umbilical Cord Blood Stem Cells through Stemness-Related Gene Regulation
Source: ACS Omega. 2026 Apr 3;11(15):22571–80. doi: 10.1021/acsomega.5c08975 (PMC13103761; doi:10.1021/acsomega.5c08975)
Supplement: Supplementary file 1 [file ao5c08975_si_001.pdf]

Supporting Information

14-Deoxy-11,12-didehydroandrographolide Promotes Ex Vivo Expansion of Umbilical Cord Blood Stem Cells through Stemness-Related Gene Regulation

Laongthip Ruknarong[¥†], Nareerat Sutjarit[ψ†], Nittaya Boonmuen[Ŧ], Puckjira Iaocharoen[γ], Tippawan Duangsong[#], Wachirachai Pabuprapap[#], Apichart Suksamrarn[#], Duangrat Tantikanlayaporn[¥, €]*

[¥]Center of Excellence in Stem Research and Innovation, Faculty of Medicine, Thammasat University, Pathum Thani 12120, Thailand

[ψ]Nutrition Unit, Faculty of Medicine Ramathibodi Hospital, Mahidol University, Bangkok 10400, Thailand

[Ŧ]Department of Physiology, Faculty of Science, Mahidol University, Bangkok 10400, Thailand

[γ]Department of Obstetrics and Gynecology, Faculty of Medicine, Thammasat University, Pathum Thani 12120, Thailand

[#]Department of Chemistry and Center of Excellence for Innovation in Chemistry, Faculty of Science, Ramkhamhaeng University, Bangkok 10240, Thailand

[€]Division of Cell Biology, Faculty of Medicine, Thammasat University, Pathum Thani 12120, Thailand

[†]These authors equally contributed to this work

*Corresponding Author: Duangrat Tantikanlayaporn

Duangrat Tantikanlayaporn

Division of Cell Biology

and Center of Excellence in Stem Research and Innovation,

Faculty of Medicine, Thammasat University, Pathum Thani 12120, Thailand

E-mail address: dkanlayaporn@gmail.com

Table of Content **Page**

Figure S1. ^{1}H NMR spectrum (400 MHz) of 14-deoxy-11,12-didehydroandrographolide (14-DDA) $CDCl_3$+5 drops CD_3OD. 3

Figure S2. ^{13}C NMR spectrum (100 MHz) of 14-deoxy-11,12-didehydroandrographolide (14-DDA) in $CDCl_3$+5 drops CD_3OD 4

Figure S3. HR-TOFMS (ES^+) spectrum of 14-deoxy-11,12-didehydroandrographolide (14-DDA) 4

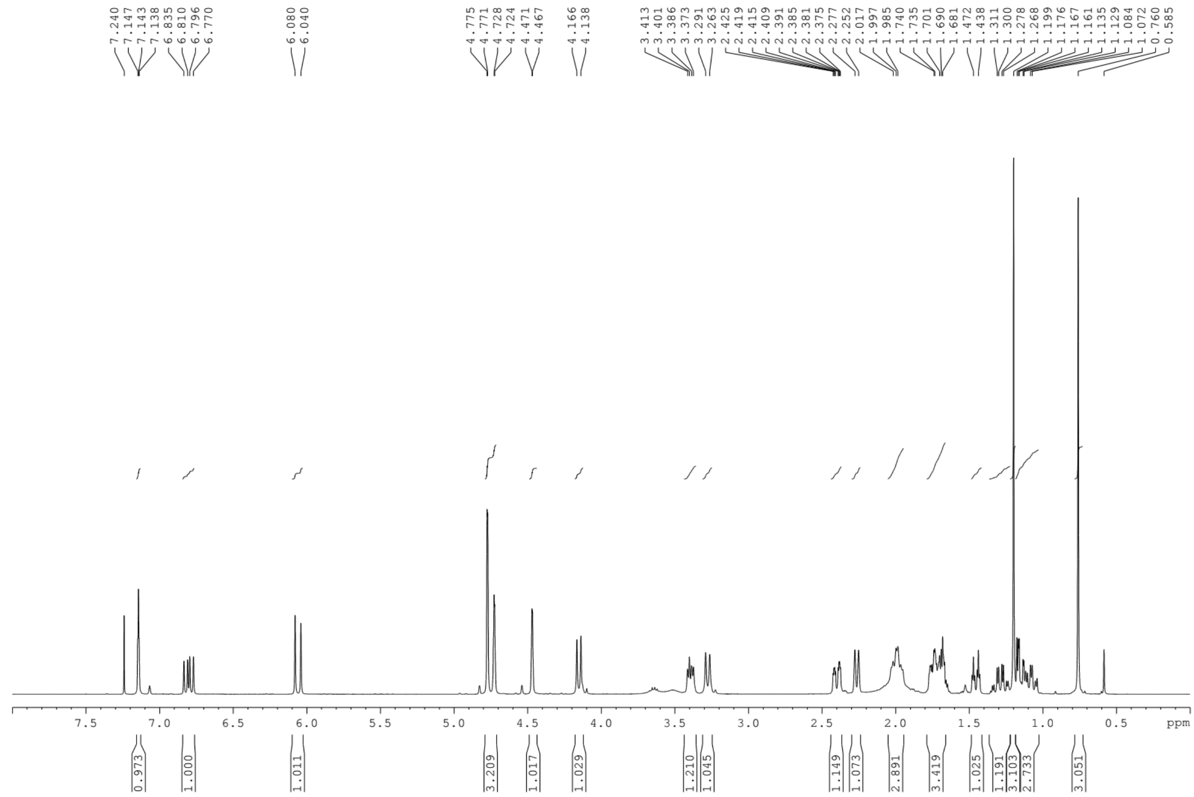

Figure S1. ^{1}H NMR spectrum (400 MHz) of 14-deoxy-11,12-didehydroandrographolide (14-DDA) in $CDCl_3$+5 drops CD_3OD.

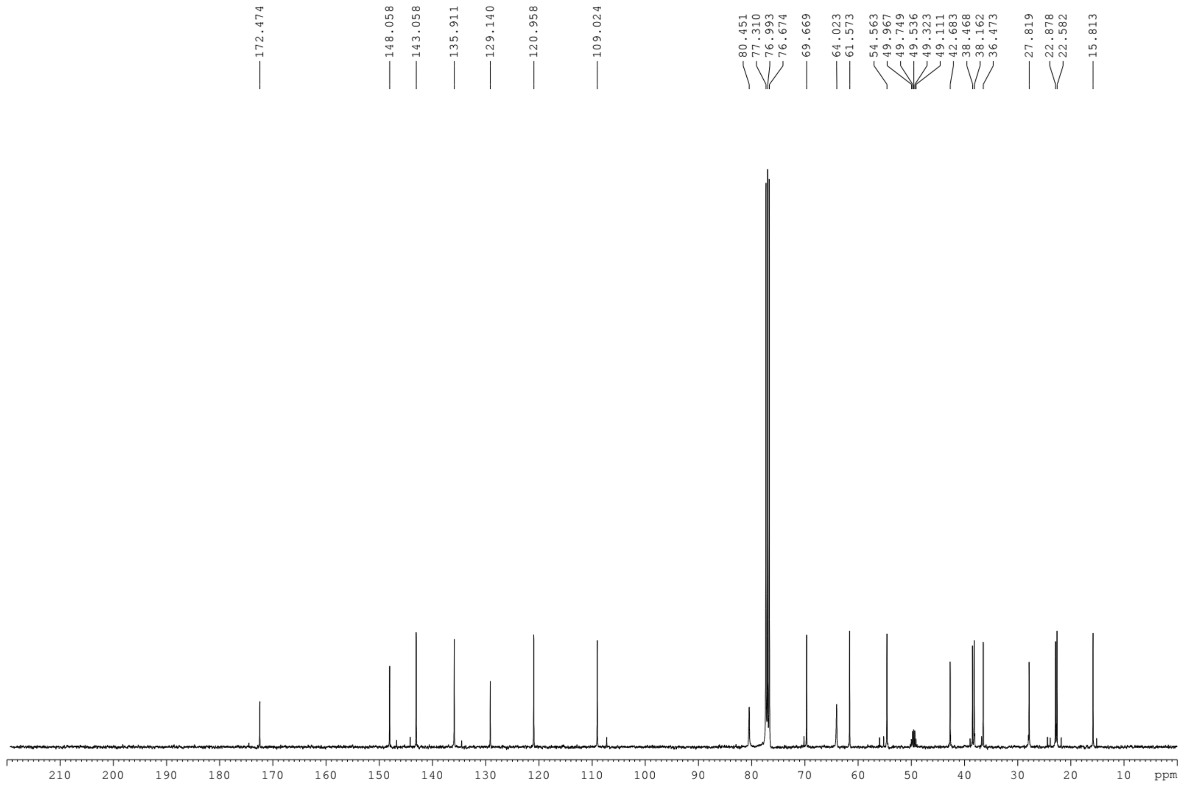

Figure S2. ^{13}C NMR spectrum (100 MHz) of 14-deoxy-11,12-didehydroandrographolide (14-DDA) in $CDCl_3$+5 drops CD_3OD.

Acquisition Parameter

Source Type	ESI	Ion Polarity	Positive	Set Nebulizer	2.0 Bar
Focus	Not active			Set Dry Heater	200 °C
Scan Begin	50 m/z	Set Capillary	4500 V	Set Dry Gas	8.0 l/min
Scan End	2000 m/z	Set End Plate Offset	-500 V	Set Divert Valve	Waste

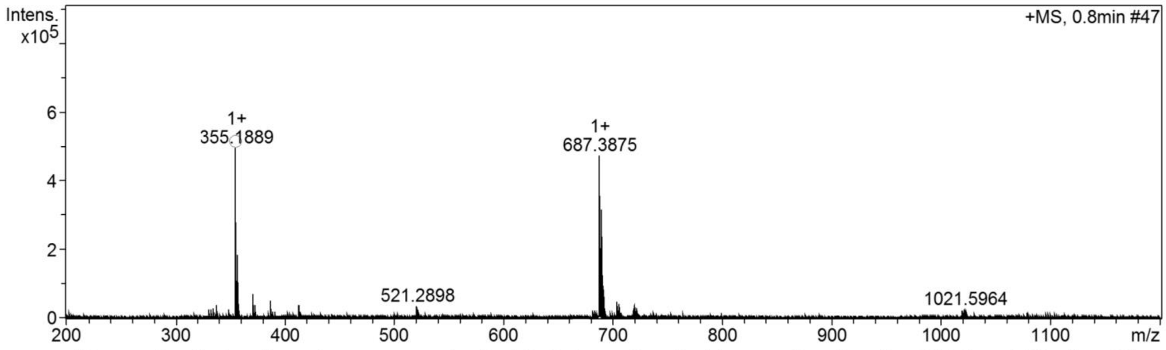

Meas. m/z	#	Ion Formula	m/z	|err| [mDa]	err [ppm]	Mean err [ppm]	rdb	N-Rule	e⁻ Conf	mSigma
355.188924	1	C20H28NaO4	355.187980	0.9	2.7	-7.9	6.5	ok	even	199.5

Figure S3. HR-TOFMS (ES^+) spectrum of 14-deoxy-11,12-didehydroandrographolide (14-DDA)
